# *Cryptosporidium* Species and Subtypes and Clinical Manifestations in Children, Peru

**DOI:** 10.3201/eid1410.071273

**Published:** 2008-10

**Authors:** Vitaliano A. Cama, Caryn Bern, Jacqueline Roberts, Lilia Cabrera, Charles R. Sterling, Ynes Ortega, Robert H. Gilman, Lihua Xiao

**Affiliations:** Centers for Disease Control and Prevention, Atlanta, Georgia, USA (V.A. Cama, C. Bern, J. Roberts, L. Xiao); Asociacion Benefica Proyectos en Informática, Salud, Medicina y Agricultura, Lima, Peru (L. Cabrera); University of Arizona, Tucson, Arizona, USA (C.R. Sterling); University of Georgia, Griffin, Georgia, USA (Y. Ortega); The Johns Hopkins University Bloomberg School of Public Health, Baltimore, Maryland, USA (R.H. Gilman)

**Keywords:** Cryptosporidium, cryptosporidiosis, pediatric, diarrhea, virulence, clinical presentations, genotype, subtype, molecular epidemiology, research

## Abstract

To determine whether clinical manifestations are associated with genotypes or subtypes of *Cryptosporidium* spp., we studied a 4-year longitudinal birth cohort of 533 children in Peru. A total of 156 infection episodes were found in 109 children. Data from first infections showed that *C. hominis* was associated with diarrhea, nausea, vomiting, general malaise, and increased oocyst shedding intensity and duration. In contrast, *C. parvum*, *C. meleagridis*, *C. canis*, and *C. felis* were associated with diarrhea only. *C. hominis* subtype families were identified (Ia, Ib, Id, and Ie); all were associated with diarrhea. Ib was also associated with nausea, vomiting, and general malaise. All *C. parvum* specimens belonged to subtype family IIc. Analysis of risk factors did not show associations with specific *Cryptosporidium* spp. genotypes or subtypes. These findings strongly suggest that *Cryptosporidium* spp. and subtypes are linked to different clinical manifestations in children.

## Abstract 2

One-sentence summary for table of contents: Different genotypes and subtypes are linked to different clinical manifestations.

Cryptosporidiosis is often observed as a pediatric disease in areas where *Cryptosporidium* spp. are endemic. Children <2 years of age are frequently infected in theses areas in community (*1**–**4*) and hospital (*5*) settings. The spectrum of symptoms is diverse, ranging from acute diarrhea, severe chronic diarrhea (*6*), or vomiting to asymptomatic infections (*2**,**3*). In community-based studies in Peru, ≈30% of immunocompetent children with cryptosporidiosis reported diarrhea (*2**,**7*). In AIDS patients, the diversity of symptoms has been linked to immune status; severe chronic diarrhea affects patients whose CD4+ counts are <200 cells/mm^3^ (*8*). A recent study in HIV-infected patients in Peru showed that only 38% with *Cryptosporidium* infections had diarrhea (*9*), although 64% of participants had CD4+ counts <200 cells/mm^3^. However, the cause for these variations is not clearly understood.

The use of molecular tools in epidemiologic investigations has provided new insights into the diversity of *Cryptosporidium* spp. infecting humans and animals (*10*). There are at least 16 established *Cryptosporidium* spp. and >40 unnamed genotypes that are potentially different species. At least 8 of them have been reported in humans: *C*. *hominis*, *C*. *parvum*, *C*. *meleagridis*, *C*. *felis*, *C*. *canis*, *C*. *muris*, and *C*. *suis*, and the *Cryptosporidium* cervine genotype. Molecular characterization of the 60-kDa glycoprotein (GP60) gene of *C*. *hominis* and *C*. *parvum* has enabled further division into subtype families and subtypes (*11*).

Humans are most frequently infected with *C*. *hominis* and *C*. *parvum* (*7**,**11**,**12*); recent reports indicate possible associations between these 2 organisms and different clinical manifestations. In Brazil, children infected with *C*. *hominis* had increased parasite shedding, more frequent presence of fecal lactoferrin, and delayed growth when compared with those infected with *C*. *parvum* (*13*). In a study of sporadic cryptosporidiosis in the United Kingdom, illness was more severe in persons infected with *C*. *hominis* than in those infected with *C*. *parvum* (*14**,**15*). A recent study reported different clinical manifestations among *Cryptosporidium* spp. in HIV-positive persons, and *C*. *hominis* was linked to more severe symptoms. The high virulence of *C*. *hominis* was evident within its subtype family Id, while absent in subtype families Ia and Ie (*16*).

In this study, we analyzed the diversity of *Cryptosporidium* at the species, subtype family, and subtype levels in children living in an area with endemic cryptosporidiosis. We also analyzed the association between clinical manifestations and infections with specific *Cryptosporidium* spp. and *C*. *hominis* subtype families.

## Methods

### Study Design

Specimens and data were obtained from a longitudinal birth cohort study of diarrheal diseases conducted during 1995–1998 in Pampas de San Juan de Miraflores, Lima, Peru. This community was initially settled in the 1980s by immigrants from rural areas. It is located in the outskirts of Lima and had at the time of the study ≈40,000 inhabitants. In this community, the prevalence of HIV infection was <1% (*2**,**7*). The study protocol was reviewed and approved by the institutional review boards of Johns Hopkins University and the Centers for Disease Control and Prevention. All participants provided informed consent before participation in the study.

### Microscopy

The study participants were asked to provide weekly fecal specimens for microscopic detection of ova and parasites, including *Cryptosporidium* spp. Stool specimens were washed and concentrated by using the modified Ritchie formalin-ether method and examined for *Cryptosporidium* spp. oocysts by microscopy of smears stained with a modified acid-fast stain. Intensity of *Cryptosporidium* spp. oocyst shedding in stools was determined by counting the number of oocysts per 50 μL of concentrated sample. We used a 0 to 3+ scoring system in which 0, negative; 1+, 1–50 oocysts; 2+, 51–150 oocysts; and 3+, >150 oocysts.

### Genotyping and Subtyping

*Cryptosporidium* spp. were identified by using a small subunit rRNA-based PCR–restriction fragment length polymorphism genotyping tool (*7**,**12**,**17*). Subtyping of *C*. *hominis* and *C*. *parvum* was based on sequence analysis of GP60 genes (*18*). Each specimen was analyzed by either method at least twice. Subtype families within *C*. *hominis* and *C*. *parvum* were determined on the basis of sequence differences in the nonrepeat region of the gene. Within each subtype family, subtypes differed from each other, mostly in the number of serine-coding trinucleotide repeats (TCA, TCG, or TCT microsatellite) located in the 5′ region of the gene. The previously established nomenclature system was used to differentiate subtypes within each subtype family (*11**,**16**,**17*). For *C*. *parvum* subtype family IIc, the original GP60 sequence for *C*. *parvum* subtype family IIc (GenBank accession no. AF164491) was assigned as IIcA5G3a. Subtypes that diverged from this sequence were assigned subsequent alphabetical extensions.

### Associated Clinical Manifestations and Risk Factors

Daily information on clinical manifestations was gathered by using structured questionnaires. These data were collected by study personnel during interviews of adult caregivers of the participants. Data included relevant gastrointestinal symptoms such as abdominal pain, fever, general malaise, nausea, vomiting, number and consistency of bowel movements, and blood in stools.

Study of potential risk factors for infections was based on sanitation and socioeconomic data obtained at study enrollment. These factors included hygiene parameters (water piped inside the house and presence of flush toilets), presence of animals (dogs, chicken, ducks, guinea pigs, rabbits, parrots, and sheep), house infrastructure (sturdy walls and roof), and indirect economic indicators (house infrastructure and possession of electronic appliances).

### Definitions

For the epidemiologic and statistical analyses, we included data from eligible children who had >6 months of participation in the study and <20% noncompliance of study procedures. For the epidemiologic analyses we used the following definitions.

Duration of an infection episode was defined as an episode that started on the first date that *Cryptosporidium* spp. oocysts were microscopically detected in stools and ended on the date of the last positive stool that was followed by at least 3 weekly specimens that were microscopically negative. The length of the infection episode was the number of days between the start and end dates.

An episode of diarrhea was defined as a child having >3 liquid or semiliquid bowel movements on any day and the mother’s assessment that the child had diarrhea. Diarrhea was considered associated with an episode if it occurred within 7 days of a positive result for *Cryptosporidium* spp.

### Statistical Analysis

Statistical analyses included data from participants infected with 1 species of *Cryptosporidium* and compared children with a specific *Cryptosporidium* sp. or *C*. *hominis* subtype family with all other participants not infected with that species or subtype family. Subtype families were compared because of the extensive sequence polymorphism in the nonrepeat regions of GP60, and subtypes within families primarily differed from each other in the length of the serine stretch at the beginning of the protein. Data from the few children infected with >1 species or subtype determinations that were conflicting with genotype categorizations were excluded from that particular comparison. Because all *C*. *parvum* in this population belonged to 1 subtype family, results were presented at the species level. Few participants were infected with *C*. *canis* and *C*. *felis* and these species are genetically divergent from *C*. *hominis*, *C*. *parvum*, and *C*. *meleagridis*. Therefore, the data for these persons were pooled.

Poisson regression was used to compare incidence rates of gastrointestinal symptoms (dependent variables) and infections with *Cryptosporidium* spp. or subtype families (independent variables) detected in each infection episode. This model was used to incorporate individual incidence rates of infections and the duration that each person participated in the study. These regression analyses were conducted by using SAS Proc Genmod (SAS Institute, Cary, NC, USA) for linear models. The generalized estimating equations procedure was implemented to adjust for correlation among multiple infections for the same child. Statistical significance for a priori tests was set at α = 0.05. Whenever multiple subtypes were compared, a separate Bonferroni adjustment was used to maintain an overall experiment-wide α of 0.05.

The χ^2^ or Fisher exact tests were used to analyze any association between *Cryptosporidium* spp. or subtypes and animal contacts or socioeconomic risk factors. Pooled *t* test was used to investigate the differences in age at first infection episode among *Cryptosporidium* spp. and subtype families. All statistical analyses were performed by using SAS version 9.1 (SAS Institute).

## Results

A total of 533 children were enrolled, and their median age at enrollment was 14 days. They contributed 44,042 stool specimens for detection of enteric parasites and 324,067 child-days of clinical manifestation surveillance.

### Prevalence of Cryptosporidiosis

Data from 368 participants who met the evaluable criteria were included in the epidemiologic analyses. Cryptosporidiosis was detected by microscopy for 109 participants, for a total of 156 infection episodes. Among them, 71 children had 1 infection, 30 had 2 infections, 7 had 3 infections, and 1 had 4 infections.

### *Cryptosporidium* spp. Genotypes and Subtypes

Genotype data for *Cryptosporidium* spp. were obtained from 127 (81%) of 156 infection episodes. Among those genotyped, *C*. *hominis* (70%) was the species most frequently detected, followed by *C*. *parvum* (13%) and C. *meleagridis* (8%). In contrast, *C*. *canis* and *C*. *felis* were detected in 2% and 5% of cases, respectively (Table 1). Among 106 infection episodes with either *C*. *hominis* (89) or *C*. *parvum* (17), subtype analysis was successfully accomplished for 78 of 89 infections with *C*. *hominis* and 14 of 17 infections with *C*. *parvum*. Four subtype families were identified within *C*. *hominis*: Ia, Ib, Id, and Ie, the least frequent was Id. All infections with *C*. *parvum* belonged to subtype family IIc. Novel subtype sequences were deposited in GenBank under accession nos. EU095258–EU095267 (Table 2).

**Table 1:** Frequency of infections with *Cryptosporidium* spp. in 533 children, Peru

Species	No. (%) infection episodes	
	First	Overall
*C. hominis*	61 (64.9)	89 (70.1)
*C. parvum*	15 (16.0)	17 (13.4)
*C. meleagridis*	9 (9.6)	10 (7.9)
*C. canis*	2 (2.1)	2 (1.6)
*C. felis*	4 (4.3)	6 (4.7)
*C. hominis* and *C. parvum*	2 (2.1)	2 (1.6)
*C. canis* and *C. meleagridis*	1 (1.1)	1 (0.8)
No. genotyped	94	127
Total episodes	109	156

**Table 2:** Distribution of subtype families and subtypes of *Cryptosporidium hominis* and *C. parvum* in 533 children, Peru

Species	Subtype families	No. episodes (%)		Subtype: no. (%) within subtype family	GenBank accession no.
		At first infection	All		
*C. hominis*	Ia	15 (24.6)	21 (26.9)	IaA11R4: 3 (14)	EU095258*
				IaA12R4: 7 (33)	EU095259*
				IaA13R4: 1 (5)	EU095260*
				IaA13R7: 1 (5)	EU095261*
				IaA14R6: 5 (24)	EU095262*
				IaA15R3: 3 (14)	EU095263*
	Ib	16 (26.2)	23 (29.5)	IbA10G2: 23 (100)	AY262031
	Id	7 (11.5)	12 (15.4)	IdA10: 9 (75)	EU095264*
				IdA15: 1 (8)	DQ280498
				IdA20: 2 (16)	EU095265*
	Ie	15 (24.6)	19 (24.4)	IeA11G3T3:19 (100)	DQ665689
	lb + le	1 (1.6)	1 (1.3)	1 (1.3)	
	Ib + id	1 (1.6)	1 (1.3)	1 (1.3)	
	Id + ie	1 (1.6)	1 (1.3)	1 (1.3)	
*C. hominis* and *C. parvum*	Id + IIc		1		
*C. parvum*	IIc	14 (100)	14 (100)	IIcA5G3a: 12 (86)	AY738195
				IIcA5G3b: 1 (7)	EU095266*
				IIcA5G3c: 1 (7)	EU095267*

*From this study.

Several subtypes were found within subtype families Ia and Id of *C*. *hominis* and IIc of *C*. *parvum*. Subtype family Ia was the most diverse with 6 subtypes, followed by subtype families Id and IIc, each with 3 subtypes. In contrast, subtype families Ib and Ie each had only 1 subtype: IbA10G2 was the only subtype in subtype family Ib and IeA11G3T3 was the only subtype in subtype family Ie (Table 2).

### *Cryptosporidium* spp. and Oocyst Shedding

The mean age for first infections was 1.6 years of age (median 1.4 years, range 0.2–4.7 years). Infections with *C*. *parvum* occurred at a younger age than those with other genotypes, and infections with *C*. *canis* or *C*. *felis* occurred in older children. However, these differences were not statistically significant after the Bonferroni correction (Table 3).

**Table 3:** Age at first infection by *Cryptosporidium* spp. and subtype family in 533 children, Peru

Species or subtype family	No. episodes	Age, y, mean (range)	p value
*C. hominis*	61*	1.93 (0.19–9.51)	0.026†
Subtype family Ia	15	2.13 (0.67–8.05)	0.113
Subtype family Ib	16	1.38 (0.60–2.82)	0.176
Subtype family Id	7	1.41 (0.19–3.34)	0.645
Subtype family Ie	15	1.81 (0.25–9.51)	0.723
*C. parvum*	15	1.22 (0.44–2.49)	0.034†
*C. meleagridis*	9	1.43 (0.78–2.75)	0.615
*C*. *canis* or *C. felis*‡	6	2.26 (0.68–3.74)	0.039†
Mixed infections	2	1.62 (1.44–1.79)	Not done

*Eight *C. hominis* infections did not have subtype family data. †Not significant after Bonferroni adjusted α = 0.05/5 = 0.01. ‡Includes 1 mixed infection with *C. meleagridis* and *C. canis*.

The mean duration of the first infection episode was 8.1 days (median 5.5 days, range 1–40 days). Infections with *C*. *hominis* (mean 10.3 days) lasted longer than infections with other species of *Cryptosporidium* (mean 5.8 days; p = 0.001). The length of the infection episodes among children infected with different subtype families of *C*. *hominis* was not significantly different (9.3, 13.1, 7.7, and 12.8 days for Ia, Ib, Id, and Ie, respectively).

Similar patterns were observed for intensity of parasite excretion. Children infected with *C*. *hominis* had higher parasite excretion scores (mean 1.93) than those infected with other species of *Cryptosporidium* (mean 1.42; p = 0.021). Among children infected with different subtype families of *C*. *hominis*, the intensity of parasite shedding was similar.

### Sequential *Cryptosporidium* spp. Infections

Among children with complete genotyping data, sequential infections were detected in 17 children: 15 had 2 episodes of *Cryptosporidium* spp. infection and 2 had 3 episodes (total of 19 reinfection events). The median interval between infections was 10 months (range 2.1–26 months). The same *Cryptosporidium* sp. was detected in 6 of 15 children with 2 episodes and 1 of 2 children with 3 infections, all involving *C*. *hominis* (Table 4). When analysis of reinfections included *C*. *hominis* subtype family data, only 2 sequential infections occurred with the same subtype family: child 5395 had *C*. *hominis* subtype family Id in the first and second infections, and child 5076 had *C*. *hominis* subtype family Ie in the second and third episodes of cryptosporidiosis.

**Table 4:** *Cryptosporidium* spp. and subtype families of *C. hominis* detected in reinfection events in 533 children, Peru

Event	Infection		
	First	Second	Third
5444	*C. parvum* (IIc)	*C. hominis* (Id and Ie)	*C. hominis* (Ib)
5076	*C. hominis* (Id)	*C. hominis* (Ie)	*C. hominis* (Ie)*
E392	*C. hominis* (Ib)	*C. hominis* (Ie)	
K283	*C. hominis* (Ib)	*C. hominis* (Id)	
5395	*C. hominis* (Id)	*C. hominis* (Id)*	
5125	*C. hominis*	*C. hominis* (Ia)	
D037	*C. hominis*	*C. hominis* (Ia)	
5492	*C. hominis*	*C. hominis* (Id)	
5471	*C. hominis* (Ib)	*C. parvum*	
5399	*C. hominis* (Ie)	*C. felis*	
5370	*C. parvum*	*C. hominis* (Id)	
5266	*C. meleagridis*	*C. hominis* (Ib)	
H131	*C. meleagridis*	*C. hominis* (Ia)	
5082	*C. meleagridis*	*C. hominis*	
5300	*C. felis*	*C. hominis*	
5085	*C. canis*	*C. hominis*	
5049	*C. hominis* and *C. parvum*	*C. felis*	

*Reinfections with the same subtype family.

### *Cryptosporidium* spp. and Subtypes and Associated Clinical Manifestations

Distribution of species and subtype families at first infection among 109 *Cryptosporidium* spp.–infected children was similar to the distribution in all infection episodes. A second model analyzed the data from all infection episodes (Table 5).

**Table 5:** Associations between infections with *Cryptosporidium* spp. or *C. hominis* subtype families and clinical manifestations expressed as incidence rate ratios in 533 children, Peru*

Clinical manifestation	First infection			All infections	
	IRR	p value		IRR	p value
Children infected with *C. hominis* vs. those with cryptosporidiosis but not infected with *C*. *hominis*					
Nausea	5.469	<0.001†		3.531	0.037‡
Vomiting	2.252	<0.001†		2.359	0.023‡
General malaise	2.523	<0.001†		2.071	0.035‡
Diarrhea	3.690	<0.001†		4.886	<0.001†
Children infected with *C. parvum* vs. those with cryptosporidiosis but not infected with *C*. *parvum*					
Diarrhea	3.249	<0.001†		4.562	<0.01†
Children infected with *C. meleagridis* vs. those with cryptosporidiosis but not infected with *C. meleagridis*					
Diarrhea	2.484	0.006†		7.684	<0.001†
Children infected with *C. canis* or *C. felis* vs. those with cryptosporidiosis but not infected with these species					
Diarrhea	3.122	0.002†		1.528	0.662
Children infected with *C. hominis* subtype family Ia vs. those not infected with that subtype family					
Nausea	5.020	<0.001†		3.203	0.183
Vomiting	2.280	0.013‡		2.100	0.141
Diarrhea	2.442	<0.001†		3.061	0.002†
Children infected with *C. hominis* subtype family Ib vs. those not infected with that subtype family					
Nausea	12.516	<0.001†		8.402	0.006†
Vomiting	4.752	<0.001†		4.868	0.004†
General malaise	4.939	<0.001†		4.139	0.006†
Diarrhea	5.510	<0.001†		6.506	<0.001†
Children infected with *C. hominis* subtype family Id vs. those not infected with that subtype family					
Diarrhea	2.999	<0.001†		3.171	0.022‡
Children infected with *C. hominis* subtype family Ie vs. those not infected with that subtype family					
General malaise	1.830	0.010‡		1.613	0.333
Diarrhea	3.117	<0.001†		4.160	0.001†

*Determined by Poisson regression analyses. IRR, incidence rate ratio. †Statistically significant at Bonferroni corrected α = 0.01. ‡Statistically significant at p<0.05.

On the basis of microscopy results, 36% of infected children had diarrhea, 28.4% had general malaise, 16.5% had abdominal pain, 15.7% had vomiting, and 7.9% had nausea. None of the study participants reported fever or blood in stools. Overall, 44.1% reported >1 of the manifestations assessed in the study.

Associated clinical manifestations at first infection varied among different *Cryptosporidium* spp. First infections with *C*. *hominis* were associated with nausea, vomiting, general malaise, and diarrhea (Table 5). In contrast, infections with other species were associated with diarrhea only.

Patterns of clinical manifestations also varied among *C*. *hominis* subtype families. Infections with subtype family Ib were associated with nausea, vomiting, general malaise, and diarrhea. Infections with other subtype families (Ia, Id, and Ie) were generally associated with diarrhea only. A similar trend was also seen in the cumulative analysis of all infection episodes at the species and subtype family levels. A possible exception was *C*. *hominis* subtype family Ia, which showed an association with nausea and vomiting at first infections but did not show such an association in the cumulative analysis of all infection episodes (Table 5).

## Discussion

Rates of clinical manifestations in our study were lower than rates reported for a birth cohort in Brazil, where 81% of 42 participants infected with *C*. *hominis* or *C*. *parvum* had diarrhea (*13*). This difference can be attributed to differences in study designs. Our study analyzed weekly stool samples for the presence of *Cryptosporidium* spp. and other parasites in a cohort of healthy children. In contrast, the cohort study in Brazil was designed to identify causes of diarrhea, and the specimens were collected within 2 weeks of clinical identification of diarrhea.

*C*. *hominis* was the predominant species in this community-based longitudinal study, followed by *C*. *parvum* (*7*). This predominance of *C*. *hominis* has been observed in persons in other developing countries, such as pediatric populations from Malawi (*19*), Kenya (*20*), India (*21*), Haiti (*22*), and Brazil (*13*), children and elderly persons from South Africa (*23*), and hospitalized HIV-infected children from South Africa and Uganda (*24**,**25*). As reported in previous studies (*21**,**24**,**26**,**27*), we also detected few concurrent infections with multiple *Cryptosporidium* spp. or *C*. *hominis* subtype families.

We observed a comparatively large proportion of participants infected with *C*. *meleagridis*, a finding that was also reported at a high frequency in HIV-infected adults in Lima, Peru (*12**,**16*). This species has been rarely reported for studies from other locations such as Portugal (*28*), India (*21**,**26**,**29*), Taiwan (*30*), or Iran (*31*) that included either children or adults with or without HIV infections. It should be noted that the diversity of *Cryptosporidium* spp. is also affected by the methods used. We used a genotyping tool proven to distinguish several dozen species and genotypes. However, methods based on genes coding for a 70-kDa heat-shock protein (*32*), *Cryptosporidium* spp. oocyst wall protein (*33*), or a smaller fragment of the small subunit rRNA gene (*34*) discriminate fewer *Cryptosporidium* spp. and genotypes.

Overall, distribution of species and *C*. *hominis* subtype families in our study was similar to that found in an HIV study in Lima, Peru (*12**,**16*). These 2 studies were conducted in the same area but in different study populations. In both studies, all *C*. *parvum* specimens belonged to subtype family IIc, which is considered anthroponotic in origin (*17*). The normally zoonotic subtype family IIa was not seen in our study population. This finding is also supported by our risk factor data, which showed the lack of bovines in the study households and the absence of cattle farms in or near the community of Pampas de San Juan. The similarity of the species and subtype distribution in both studies is highly suggestive that the prevalence of *Cryptosporidium* spp. and subtypes in a specific location is independent of the immune status of the study population.

The role of parasite genetics in clinical manifestations of cryptosporidiosis is not clear. Studies of human volunteers showed that exposure provided some degree of protection against infection and illness; the infection rates and frequencies of infection-associated clinical manifestations were lower for subsequent infections (*35*). Thus, clinical manifestations caused by parasite differences would be better observed in primary infections. Our longitudinal birth cohort study enrolled children at an early age (median 14 days), which enabled us to study genotypes and subtypes present at first infections and their associations with different clinical manifestations.

First infections with all species and *C*. *hominis* subtype families were associated with diarrhea. However, only *C*. *hominis* subtype family Ib was also associated with nausea, vomiting, and general malaise, but *C*. *hominis* subtype families Ia, Id, and Ie, and other *Cryptosporidium* spp. were not. Previously, other studies had suggested that *C*. *hominis* might be more pathogenic than other species or might induce different clinical manifestations (*13**,**15**,**21*). Our results indicate that within *C*. *hominis*, subtype family Ib may be more pathogenic than Ia, Id, and Ie. Subtype family Ib of *C*. *hominis* is the most frequently detected *Cryptosporidium* spp. in waterborne outbreaks of cryptosporidiosis in industrialized nations (*36*).

A previous study of cryptosporidiosis in HIV-infected persons in Peru showed that infections with different species or subtype families were associated with different clinical manifestations. Patients infected with subtype families Ib and Id of *C*. *hominis*, *C*. *parvum*, or *C*. *canis*/*C*. *felis* were more likely to have chronic diarrhea, and patients infected with *C*. *parvum* were more likely to have infection-associated vomiting (*16*). Overall, subtype family Id was the most virulent in the HIV study and was strongly associated with diarrhea in general and chronic diarrhea in particular. Subtype family Ib was also marginally associated with diarrhea and vomiting but not with chronic diarrhea. In this study, however, Id was only associated with diarrhea. This difference may be caused by the fact that chronic cryptosporidiosis, the life-threatening manifestation of the disease in AIDS patients, was never detected in this study of pediatric patients, and few children in this study were infected with subtype family Id, which might have prevented us from assessing its clinical manifestations fully. Nevertheless, our study corroborated the previous observation of defined patterns of clinical manifestations associated with different *Cryptosporidium* spp. and *C*. *hominis* subtype families.

We also conducted a risk factor analysis for predictors of infection, including age at first infection, in which we did not identify statistically significant associations between any *Cryptosporidium* spp. or subtype families and any of the variables analyzed, although they covered basic aspects of sanitation and zoonotic, foodborne, and waterborne transmission. One possible explanation is that our questionnaires did not obtain data on factors that were relevant. However, the same questionnaire successfully identified infection risk factors for other organisms in the same community (*2*). A more likely explanation is that because most *Cryptosporidium* spp. in this study were anthroponotic in origin, children may be constantly exposed to these ubiquitous parasites through different transmission routes. Therefore, single exposure variables were not identified as risk factors. This constant exposure may also fit the age distribution pattern of cryptosporidiosis in the community, in which most cases are found in children <2 years of age, occasionally found in older children, and almost never found in immunocompetent adults. This finding is in contrast to transmission of *Cryptosporidium* spp. in industrialized nations, where infections have been frequently associated with waterborne transmission from either drinking water (*37*) or recreational water (*38*).

In conclusion, clinical manifestations of cryptosporidiosis in healthy populations in disease-endemic areas are likely diverse, and the spectrum of these clinical manifestations can be attributed in part to the different species of *Cryptosporidium* and subtype families of *C*. *hominis*. Although further laboratory and longitudinal cohort studies in other disease-endemic areas are needed to validate our observations, these results demonstrate that parasite genetics may play an important role in the clinical manifestations of human cryptosporidiosis. Future studies should be conducted in different geographic settings; they should overcome some potential limitations of this study, such as lack of data on other gastrointestinal pathogens, which might have confounded the clinical findings, and small sample sizes, which had limited the power of the statistical analyses.
